# Fetal Biometric Parameter Reference Charts of a Central Anatolian Turkish Population

**DOI:** 10.7759/cureus.55252

**Published:** 2024-02-29

**Authors:** Ozlem Dulger, Figen Taser, Usame O Osmanoglu, Aliye N Serin

**Affiliations:** 1 Department of Obstetrics and Gynecology, Faculty of Medicine, Karamanoglu Mehmetbey University, Karaman, TUR; 2 Department of Anatomy, Faculty of Medicine, Karamanoglu Mehmetbey University, Karaman, TUR; 3 Department of Biostatistics, Faculty of Medicine, Karamanoglu Mehmetbey University, Karaman, TUR

**Keywords:** intrauterine growth, obstetrics ultrasound, fetal biometric parameters, femur length, abdominal circumference, head circumference, biparietal diameter, fetal morphometry, fetal growth charts, fetal biometry

## Abstract

Purpose: The assessment of fetal biometry using ultrasound provides accurate pregnancy dating and also screening of fetal growth. Fetal biometry, which is common practice in the second and third trimesters of pregnancy, is fetal morphometry, which involves taking measurements of the different anatomical body parts. These fetal dimensions vary on ethnicity. The aim of this study is to demonstrate fetal biometric parameters measurement results of the Central Anatolia Turkish population with detailed percentile tables and graphs to screen fetal growth more accurately.

Methods: This cross-sectional study was performed on a total of 1132 fetuses (47% girl, and 53% boy) between 15 and 40 weeks of gestation. Biparietal diameter (BPD), head circumference (HC), abdominal circumference (AC), and femur length (FL) measurements are performed in a standardized manner every gestational week. BPD and HC were measured at the level of the thalami on the horizontal plane of the fetal head. HC was measured using the ellipse method. AC measurement was taken at the circular cross-section of the upper fetal abdomen. FL was measured along with the ossified diaphysis of the femur. All measurements were taken in millimeters.

Results: Pregnant women’s mean age was 27.58 (17-43), and the mean body mass index was 27.68 (15.06-50.78) as demographic data. 38.13% of women had their first, 29.74% had their second, and 32.13% had three or more gestations within our study. Percentile data of fetuses for each parameter (BPD, HC, AC, and FL) and for each week were shown as tables and percentile graphics. Fetal 50th percentile measurements were compared between our study and other studies from different countries. The Kruskal-Wallis test results showed that BPD (p = 0.827), HC (p = 0.808), AC (p = 0.846), and FL (p = 0.725) values have a statistically similar mean in all studies. Hierarchical cluster analysis results showed that our results for BPD, HC, AC, and FL percentile curves have been found closer to Italian population results. However, our results were statistically different from Asian, Nigerian, non-Hispanic American, and Brazilian populations for each of the different parameters.

Conclusion: The specialization of fetal biometric charts for a particular population can ensure a more accurate assessment of fetal growth rate. We showed fetal biometric percentile tables and graphics of the Central Anatolian Turkish population in this study. These results may provide a valuable contribution to obstetrical practice. Further studies can be conducted in different regions of Turkiye, thus comparisons could be possible over the country.

## Introduction

Assessment of fetal biometry using ultrasound, which provides accurate pregnancy dating and also screens fetal growth, is a very important part of routine examination in the second and third trimesters of pregnancy for prenatal care [1, 2]. Fetal biometry is fetal morphometry with measurements of the different anatomical body parts, including biparietal diameter (BPD), head circumference (HC), abdominal circumference (AC), and femur length (FL) [1, 2]. Serial measurements of multiple parameters are needed in the evaluation of fetal growth [1, 2].

The evaluation of fetal biometry is usually performed via the comparison of measured values with estimated values of computer programs, which are derived mainly from reference charts or equations [1-5]. Gestational age and fetal weight can be estimated using these morphologic measurements [1-5]. Thus, fetal growth can be evaluated during overall gestation. These biometric measurements can be combined to find the estimated fetal weight (EFW) using various formulas to provide a clinically relevant estimate of fetal growth [3-5]. Hadlock et al. determined a widely used equation for EFW using HC, AC, and FL [3, 4]. The observed EFW and standard EFW were compared, and individual fetal size could be evaluated using this method. Programs on USG devices were developed upon these formulas [4, 5]. 

Besides ethnicity, genetics and environmental factors such as maternal habits, nutrition, parity, socioeconomic status, and diseases of a particular population affect fetal development [1, 5, 6].

Measurements are mostly compared to standard reference charts to evaluate fetal biometry within the normal distribution of the reference population. This situation can lead to incorrect evaluations. Therefore, we would like to measure and showcase our population's fetuses' normal dimensions in detail within this study. This study aims to define fetal biometric parameters and make our own percentile tables and graphics for the Central Anatolian Turkish population. Also, we aimed to compare these results with other local populations. Therefore, comparisons and differences from other ethnic populations were considered very important for this study.

## Materials and methods

This cross-sectional study population consisted of 1132 uncomplicated singleton pregnant volunteer women who came for routine ultrasound examination to Karamanoglu Mehmetbey University-Karaman Training and Research Hospital (Karaman, Turkiye) between April 2022 and March 2023 for a 12-month period. The study was approved by the ethics committee, and before the inclusion of the patient, informed consent was obtained (approval date:08.03.2022, protocol ID: 2022-KAEK-154-02-12).

The study was designed prospectively to evaluate normal reference charts for fetal ultrasound measurements. Four biometric parameters, which consist of fetal biparietal diameter (BPD), head circumference (HC), abdominal circumference (AC), and femur length (FL), were measured between the 15th and 40th weeks of gestation. All measurements were made by two obstetrician medical doctors (OD, ANS) with experience in sonography.

Gestational age was based on the last menstrual period, with a cycle length of 26-30 days, and was confirmed by ultrasound before the 15th week of gestational age. Pregnant women in the range of 15-40 weeks were included in the study. Known abnormal karyotypes or congenital malformations were considered exclusion criteria. Also, pregnant foreigners residing in the country were not included in the study to make the charts accurate and ethnicity-sensitive. Pregnant women with concomitant chronic diseases possibly affecting fetal growth, including diabetes mellitus, asthma, hypertension, renal disease, and thyroid disease, were not included. Pregnants with a history of obstetric complications, intrauterine growth deficiency, or macrosomia were not included, too.

BPD, HC, AC, and FL measurements were taken in a standardized style depending on the required criteria for obtaining each measurement [1, 2, 5, 7]. All measurements were taken in millimeters.

BPD was measured on the horizontal plane of the fetal head at the level of the thalami. BPD measurement was performed as the widest diameter perpendicular to the midline (falx cerebri) from the outer edge of the upper parietal bone to the inner edge of the lower one [1, 2, 5, 7].

HC measurement was also taken at the level of the thalami on the horizontal plane using the ellipse method [1, 2, 5, 7].

AC was measured on the transverse section of the upper fetal abdomen. The measurement was taken at the circular cross-section of the abdomen, visualizing the spine, stomach bubble, and intrahepatic portion of the umbilical vein at the level of the portal sinus, one full rib on each lateral side [1, 2, 5, 7].

FL was measured along with the ossified diaphysis without including the distal femoral epiphysis. The whole femur diaphysis was displayed on the screen to optimize the measurement of the FL [1, 2, 5, 7].

SPSS v. 25.0 (IBM Corp., Armonk, NY) was used for statistical analysis and graphics. Percentile curves were created with 5th, 10th, 25th, 50th, 75th, 90th and 95th percentile values. Shapiro-Wilks normality test and Kruskal-Wallis H test were applied to analyze the data set. The closeness of the percentile values of the studies was determined by hierarchical cluster analysis. Results were shown dendrogram graphics using the Ward Linkage method. Studies with missing data could not be included in the cluster analysis. The statistical significance level was set at p<0.05.

## Results

Regarding demographic data in our study group, the pregnant women’s mean age was 27.58 (17-43) and BMI was 27.68 (15.06-50.78). Around 38.13% of women had their first, 29.74% had their second, and 32.13% had three or more gestations within our study. Around 47% of babies were girls, and 53% were boys.

Tables 1-4 indicate average fetal measurements of the 5th, 10th, 25th, 50th, 75th, 90th, and 95th percentiles between the 15th and 40th weeks of gestation for each parameter of BPD, HC, AC, and FL, respectively. Also, standard deviation values are given in these tables.

**Table 1:** Gestational week-specific percentiles for BPD (mm) GW, gestational week; P, percentile; SD, standard deviation; BPD: biparietal diameter

GW	P_5_	P_10_	P_25_	P_50_	P_75_	P_90_	P_95_	SD
15	28.3	29.2	31.2	32.7	34.30	36.6	37.5	3.5
16	31.3	32.7	33.6	35.0	36.2	38.8	40.2	3.2
17	34.2	35.3	37.4	39.0	40.5	41.5	42.3	3.1
18	37.8	39.3	40.7	42.5	43.7	45.0	46.2	3.1
19	41.3	41.8	43.4	44.7	46.3	49.1	50.0	3.4
20	43.6	46.1	47.4	49.0	50.2	51.5	53.8	3.4
21	46.4	47.1	49.7	51.5	52.7	54.4	56.7	3.8
22	48.9	50.3	52.4	54.7	56.0	57.2	58.4	3.6
23	53.5	54.1	55.9	57.9	60.3	61.7	63.4	3.8
24	56.1	57.1	58.7	60.4	62.2	63.9	66.3	3.7
25	58.8	59.4	61.1	62.9	65.5	67.4	68.9	4.0
26	61.5	62.9	65.0	67.0	69.1	70.6	71.1	3.8
27	63.9	66.0	67.5	69.3	72.6	74.8	76.9	4.8
28	68.9	69.3	70.6	72.2	75.7	77.9	78.0	3.9
29	70.2	70.6	73.2	75.9	78.2	80.9	83.3	5.0
30	71.7	73.3	74.9	79.4	81.2	82.9	84.2	4.9
31	74.6	75.5	77.7	79.5	81.3	83.9	84.3	3.9
32	74.6	77.2	79.6	81.7	84.5	87.1	88.3	5.1
33	78.6	79.0	83.1	84.3	87.7	88.4	92.0	5.0
34	81.1	82.9	85.1	86.5	88.7	90.7	93.2	4.3
35	81.9	83.9	86.5	88.6	90.4	92.6	93.5	4.4
36	82.8	85.3	87.8	90.1	92.6	94.0	95.7	4.7
37	83.8	86.3	88.2	91.2	93.2	95.1	97.2	4.4
38	86.0	87.7	89.5	91.8	93.1	96.3	98.8	5.4
39	87.8	88.4	90.8	92.4	94.6	97.0	101.3	6.2
40	89.1	90.6	93.2	95.5	96.6	99.9	103.1	6.8

**Table 2:** Gestational week-specific percentiles for HC (mm) GW, gestational week; P, percentile; SD, standard deviation; HC, head circumference

GW	P_5_	P_10_	P_25_	P_50_	P_75_	P_90_	P_95_	SD
15	103.9	107.8	113.2	118.0	123.6	129.9	131.5	10.6
16	114.5	118.6	123.20	128.8	133.1	139.7	142.7	10.6
17	128.0	129.3	136.4	141.7	145.9	149.1	149.9	9.1
18	139.0	142.7	151.0	155.8	159.1	163.6	169.2	10.9
19	151.1	154.8	160.4	163.3	171.3	176.9	178.2	10.6
20	163.8	167.9	172.8	179.2	186.4	189.8	190.9	10.8
21	174.2	177.9	182.7	187.0	193.9	197.8	206.4	11.5
22	183.0	188.0	195.8	199.7	205.2	211.2	218.8	12.6
23	199.8	201.1	208.6	215.3	220.3	226.9	231.9	12.4
24	210.4	212.2	215.3	223.5	229.8	235.8	240.3	11.8
25	220.6	223.5	227.5	233.2	239.7	248.7	252.1	12.2
26	228.2	233.5	239.4	246.9	252.3	256.3	259.3	11.8
27	235.1	240.6	248.1	254.7	265.0	270.5	275.6	15.4
28	253.1	256.9	263.8	267.6	271.9	279.8	285.2	11.6
29	260.3	264.4	271.2	277.0	283.4	287.4	291.6	11.8
30	268.7	271.7	280.0	285.0	293.8	305.0	306.8	15.2
31	274.5	278.9	283.1	290.0	296.5	301.5	308.8	12.5
32	281.5	288.2	291.3	297.2	305.0	311.0	314.5	12.3
33	296.0	300.1	303.2	305.9	313.7	319.6	326.0	10.9
34	293.4	299.7	306.8	311.7	320.4	326.2	333.2	14.4
35	303.0	307.1	314.0	319.8	325.4	330.6	339.0	12.9
36	309.3	313.6	317.8	324.3	333.5	340.4	344.6	13.6
37	308.6	314.2	321.0	328.5	338.5	345.5	354.8	16.9
38	315.1	318.0	323.8	331.6	340.9	346.9	349.4	13.9
39	321.5	325.5	331.1	336.7	344.3	349.4	356.9	12.9
40	322.9	328.4	333.1	336.5	348.2	352.6	358.7	23.0

**Table 3:** Gestational week-specific percentiles for AC (mm) GW, gestational week; P, percentile; SD, standard deviation; AC, abdominal circumference

GW	P_5_	P_10_	P_25_	P_50_	P_75_	P_90_	P_95_	SD
15	84.6	88.8	94.3	97.5	104.2	107.2	110.7	9.7
16	93.7	95	101.8	106.7	110.8	116.5	119.2	10
17	106.5	109.9	114.2	118.8	124.6	130	132.9	10.1
18	114.6	117.7	124.3	130.4	134.4	139.6	145	11.2
19	129.4	131.2	135.4	140.2	145	151.1	152.6	9.3
20	137.5	142.6	148.3	153.7	158.7	162.2	168.5	11
21	147.4	152.4	155.5	160.2	168.1	173.6	177.1	11.2
22	160.1	163	168	173.7	176.6	184	190.8	11.1
23	164.9	172.6	180.5	186.4	191.8	198.5	203.3	13.8
24	177	182.1	190.3	195.2	204.1	207.1	210	12.7
25	190.9	195.5	201.8	206.9	213.5	223.9	226.6	13.7
26	196.3	203.6	207.8	217.4	224.6	230	231.8	13.7
27	208.9	214.8	221.6	227	231.1	242.7	244.6	13.4
28	215.3	219	229.9	240.3	247.4	258.6	261	18.2
29	233.3	236.2	241.5	248.4	253.8	263.1	265.8	12.7
30	245.4	249.7	260.3	265.9	272.1	276.9	283.8	14.1
31	247.2	249.1	261.2	269.2	276.2	283.7	288.3	16.1
32	262.3	263	269.4	277.4	285.3	292.2	300.4	14.7
33	278.6	282	287.1	296.7	302	310.2	312.9	13.6
34	281.8	283	292	300.6	309	315.2	317.7	14.8
35	284	289.6	300	310.3	317.2	328.5	331.6	17.6
36	287.9	294.8	306	314.9	326.2	335.2	339.8	19.9
37	293	300.8	317.5	328.9	340.4	348.1	349	19.1
38	296.7	310	322.3	332.6	340.2	350.2	354.5	18.5
39	303.6	312.2	326.7	338.7	346.4	353.5	359.8	25.8
40	308	316.3	328.7	340.9	356.6	363.4	371.4	23.9

**Table 4:** Gestational week-specific percentiles for FL (mm) GW, gestational week; P, percentile; SD, standard deviation; FL, femur length

GW	P_5_	P_10_	P_25_	P_50_	P_75_	P_90_	P_95_	SD
15	14.1	14.8	16	18.2	19.2	21	22.5	3.2
16	17.2	17.9	19.6	20.7	21.6	23.4	24.7	2.8
17	20.3	21	22	23.9	25	26.4	27.1	2.7
18	23	23.7	25.4	27.1	29	29.9	30.8	3.1
19	25.5	26.2	26.9	29	30.7	32.2	32.5	2.9
20	30.1	30.4	31.5	32.4	33.9	35.5	37.1	2.6
21	32	32.6	33.5	34.8	36.1	37.4	37.8	2.3
22	35.3	36.4	37.1	38.1	39.4	40.4	40.9	2.1
23	36.4	38.1	39.4	40.9	42.6	43.6	44.8	3
24	39.4	40.3	41.9	43.6	45.2	46.4	46.7	2.9
25	41.9	42.6	44.1	45.5	46.7	48.3	48.7	2.7
26	44.5	45.5	47.2	48.4	50.3	51	51.4	2.7
27	46.8	48.3	49.4	50.8	52.2	54.5	56.6	3.5
28	48.1	50.7	51.9	53.6	55.4	56.6	57.5	3.4
29	51.2	53.1	54.3	55.8	57.5	58.3	59.1	2.9
30	53.3	54.6	55.9	57.3	58.5	61.3	63.1	3.6
31	55.5	56.6	58	59.4	61	61.5	61.8	2.5
32	56.9	58.6	59.7	61	62.9	64.5	65.4	3.1
33	59.4	60.6	62.3	63.9	65.7	66.7	67.9	3.2
34	62	62.5	63	65.1	66.6	67.8	69.4	2.9
35	63	63.8	65.5	67.1	68.5	70.7	72.4	3.5
36	63.6	65.2	66.7	68.2	69.8	71.8	72.9	3.4
37	64.7	66.1	67.7	70.4	72.1	74.1	74.5	3.9
38	67.2	67.9	69.8	72.3	73.9	75.5	77.3	3.8
39	69.2	70.2	71.9	73.1	75.5	77	78.6	3.5
40	70.3	71	72.9	74.1	76.1	78.8	79.1	4.4

**Figure 1:**
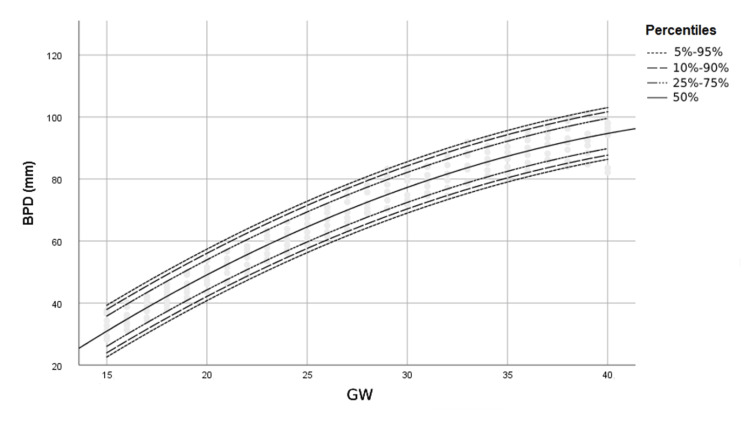
Percentiles of BPD (mm) by gestational week (GW) BPD: Biparietal diameter, GW: Gestational week

**Figure 2:**
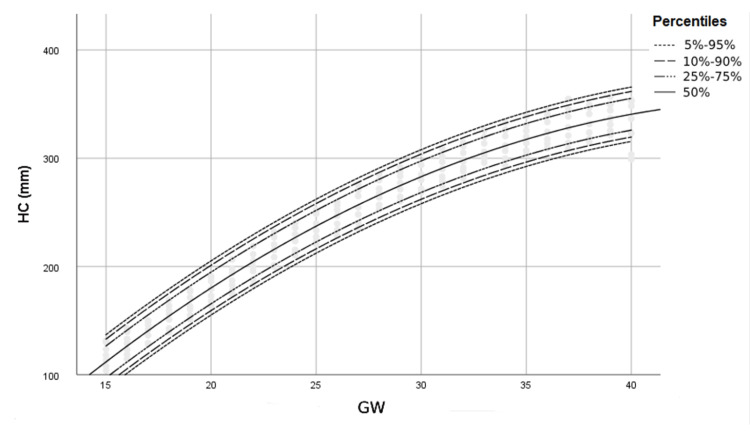
Percentiles of HC (mm) by gestational week (GW) HC: Head circumference, GW: Gestational week

**Figure 3:**
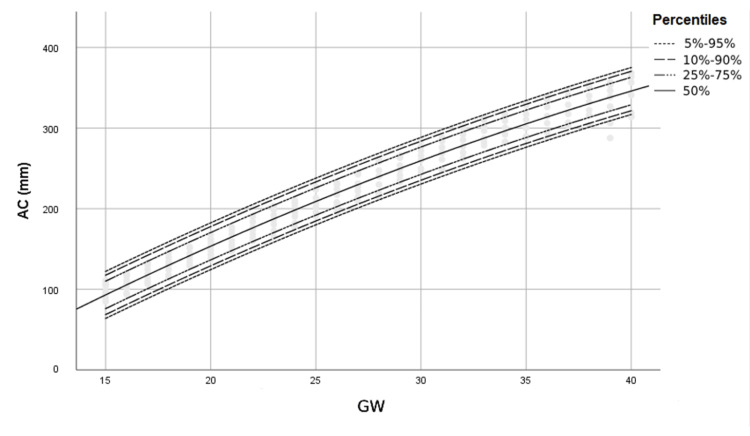
Percentiles of AC (mm) by gestational week (GW) AC: Abdominal circumference, GW: Gestational week

**Figure 4:**
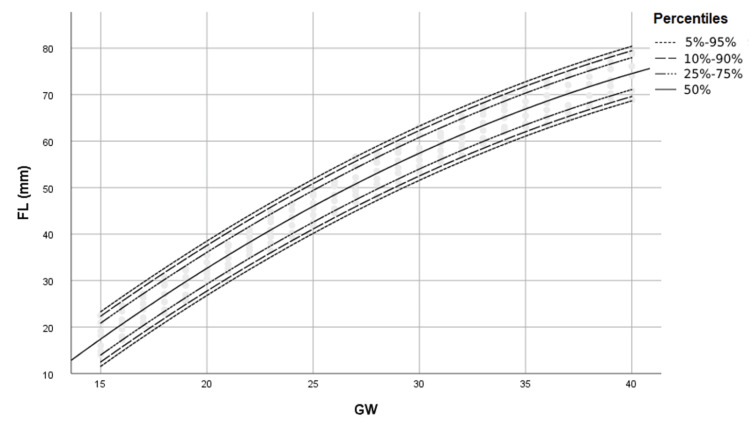
Percentiles of FL (mm) by gestational week (GW) FL: Femur length, GW: Gestational week

Figures 1-4 show percentile curves of 5th, 10th, 25th, 50th, 75th, 90th, and 95th of BPD, HC, AC, and FL values, respectively.

Tables 5-8 indicate comparative fetal median (50th percentile) measurements of the 15th to 40th weeks of gestation between our study and other studies with median values of fetal biometric parameters of BPD, HC, AC, and FL, respectively.

**Table 5:** Comparison of BPD in millimeters at the 50th percentile for each gestational week

GW	Turkish-Our Study	Brazilian(12)	Nigerian (17)	Italian (20)	NonhispanicWhite (6)	Asian(6)	Polish (19)
15	32.7	30.0	32.0	33.2	30.3	30.1	-
16	35.0	33.0	33.1	36.1	33.7	33.6	-
17	39.0	37.0	38.0	39.1	37.0	36.9	-
18	42.5	41.0	39.8	42.1	40.1	40.0	-
19	44.7	44.0	43.0	45.2	43.2	43.1	-
20	49.0	48.0	46.3	48.4	46.3	46.1	46.0
21	51.5	51.0	51.8	51.6	49.4	49.2	50.0
22	54.7	55.0	55.0	54.8	52.6	52.3	53.0
23	57.9	58.0	57.0	58.0	55.7	55.5	56.0
24	60.4	61.0	58.7	61.1	58.9	58.6	60.0
25	62.9	64.0	62.1	64.2	61.9	61.7	63.0
26	67.0	67.0	64.9	67.3	65.0	64.7	66.0
27	69.3	70.0	66.5	70.2	68.0	67.7	69.0
28	72.2	72.0	67.0	73.1	70.8	70.6	72.0
29	75.9	75.0	72.3	75.8	73.6	73.3	75.0
30	79.4	77.0	74.4	78.4	76.4	76.0	77.0
31	79.5	80.0	77.4	80.9	78.9	78.5	80.0
32	81.7	82.0	79.5	83.1	81.4	80.8	82.0
33	84.3	84.0	82.2	85.2	83.6	83.0	84.0
34	86.5	87.0	83.3	87.1	85.6	85.0	87.0
35	88.6	89.0	86.1	88.8	87.4	86.8	89.0
36	90.1	90.0	88.4	90.2	89.0	88.5	90.0
37	91.2	92.0	89.8	91.4	90.3	89.9	92.0
38	91.8	94.0	93.3	92.3	91.5	91.1	94.0
39	92.4	96.0	94.1	92.9	92.4	92.1	95.0
40	95.5	97.0	96.0	93.2	93.3	93.0	96.0

**Table 6:** Comparison of HC in millimeters at the 50th percentile for each gestational week

GW	Turkish (Our Study)	Brazilian (12)	Italian (20)	Non-Hispanic White (6)	Asian (6)	Polish (19)
15	118	105	123	111.6	110.5	-
16	128.8	119	135	124.3	123.3	-
17	141.7	132	146	136.6	135.6	-
18	155.8	146	158	148.5	147.5	-
19	163.3	159	170	160.3	159	-
20	179.2	171	182	172.2	170.7	174
21	187	183	193	184.1	182.4	185
22	199.7	195	205	196	194	196
23	215.3	206	217	207.8	205.7	207
24	223.5	217	228	219.4	217.1	218
25	233.2	227	239	230.8	228.3	229
26	246.9	238	250	241.8	239.2	239
27	254.7	247	261	252.5	249.7	249
28	267.6	257	271	262.7	259.8	259
29	277.1	265	281	272.5	269.3	269
30	285	274	290	281.8	278.3	278
31	290	282	298	290.4	286.7	286
32	297.2	290	306	298.5	294.4	295
33	305.9	297	313	305.9	301.7	302
34	311.7	304	319	312.5	308.3	309
35	319.8	310	325	318.4	314.2	315
36	324.3	317	330	323.5	319.5	321
37	328.5	322	333	327.9	324.1	326
38	331.6	328	336	331.8	327.8	330
39	336.7	332	337	335.1	330.7	334
40	336.5	337	338	338	332.6	337

**Table 7:** Comparison of AC in millimeters at the 50th percentile for each gestational week

GW	Turkish (our study)	Brazilian (12)	Italian (20)	Turkish (21)	Turkish (29)	Turkish (26)	American (30)	Non-Hispanic White (6)	Asian (6)	Polish (19)
15	97.5	91	103	99.3	106	-	-	91.2	90.4	-
16	106.7	103	114	110.9	116	-	-	103.4	102.5	-
17	118.8	114	125	122.3	121	-	-	115.6	114.5	-
18	130.4	126	136	133.7	134	-	141.3	127.7	126.3	-
19	140.2	137	148	144.9	140	-	151	139.7	138	-
20	153.7	148	159	156	156	152	151.9	151.7	149.6	149
21	160.2	159	170	167	167	164	166	163.6	161.1	160
22	173.7	170	181	177.8	176	175	177.1	175.4	172.4	171
23	186.4	181	192	188.6	189	187	190.8	187	183.4	182
24	195.2	191	203	199.2	200	198	203.1	198.4	194.3	193
25	206.9	202	214	209.7	212	209	202.6	209.6	204.9	204
26	217.4	213	224	220.1	223	219	226.3	220.6	215.4	215
27	227	223	235	230.3	234	230	229	231.4	225.8	227
28	240.3	233	245	240.5	242	240	241.5	242.3	236.1	238
29	248.4	243	255	250.5	256	250	271.8	253.2	246.7	248
30	265.9	253	265	260.4	262	260	271.8	264.3	257.3	259
31	269.2	263	275	270.2	269	269	282.2	275.4	268	270
32	277.4	273	284	279.8	283	278	288.7	286.5	278.7	280
33	296.7	283	293	289.4	293	287	298	297.5	289.1	290
34	300.6	292	302	298.8	304	296	305.6	308.3	299.3	300
35	310.3	302	310	308.1	315	305	331.1	318.8	309	309
36	314.9	311	318	317.3	327	313	346.5	328.8	318.3	318
37	328.9	321	325	326.3	332	321	362.2	338.2	327.2	326
38	332.6	330	332	335.3	345	328	350.7	346.9	335.9	334
39	338.7	339	339	344.1	357	335	359.3	354.7	344.5	342
40	340.9	348	345	352.8	362	342	373.3	361.4	353.3	349

**Table 8:** Comparison of FL in millimeters at the 50th percentile for each gestational week

GW	Turkish (our study)	Brazilian (12)	Italian (20)	Nigerian (17)	Non-Hispanic White (6)	Asian (6)	Polish (19)
15	18.2	17	18.7	18	15.8	15.7	-
16	20.7	2	21.7	2	19.3	19.1	-
17	23.9	23	24.7	24	22.6	22.4	-
18	27.1	26	27.7	25	25.7	25.4	-
19	29	29	30.6	29	28.7	28.2	-
20	32.4	31	33.4	32	31.7	31	32
21	34.8	34	36.2	36	34.6	33.8	34
22	38.1	37	38.9	37	37.4	36.5	37
23	40.9	39	41.5	4	40.1	39.1	40
24	43.6	42	44.1	42	42.7	41.7	43
25	45.5	44	46.6	45	45.2	44.2	45
26	48.4	47	49.1	48	47.5	46.6	48
27	50.8	49	51.4	47	49.8	49	50
28	53.6	51	53.7	52	52	51.2	53
29	55.8	53	55.9	54	54.1	53.4	55
30	57.3	56	58.1	56	56.3	55.5	58
31	59.4	58	60.1	58.5	58.4	57.5	60
32	61	6	62.1	61	60.4	59.5	62
33	63.9	61	64	64	62.4	61.4	65
34	65.1	63	65.8	66	64.3	63.2	67
35	67.1	65	67.5	67	66.1	65	69
36	68.2	67	69.1	7	67.7	66.7	71
37	70.4	68	70.6	72	69.3	68.3	72
38	72.3	7	72	74	70.6	69.7	74
39	73.1	71	73.4	76	71.8	71.1	75
40	74.1	73	74.6	78.5	72.8	72.4	77

The Kruskal-Wallis test was used to compare the means of continuous variables. 

According to the test results, BPD values have a statistically similar mean in all studies (p=0.827). HC values have a statistically similar mean in all studies (p=0.808). AC values have a statistically similar mean in all studies (p=0.846). FL values have a statistically similar mean in all studies (p=0.725).

**Figure 5:**
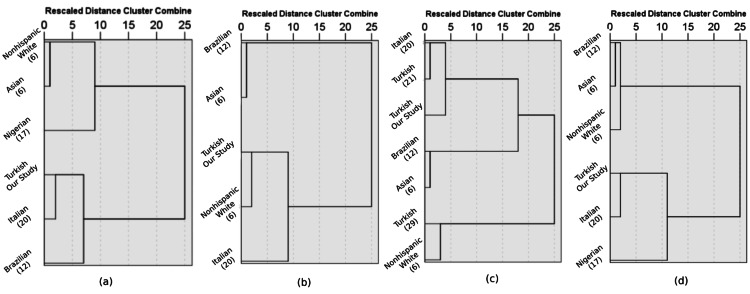
Dendrogram graphics of different studies' percentile curves according to hierarchical cluster analysis (a) BPD, (b) HC, (c) AC, (d) FL BPD: fetal biparietal diameter; HC: head circumference; AC: abdominal circumference; FL: femur length

We used Hierarchical Cluster Analysis to determine the resemblance of percentile curves of different studies as a further analysis. Results were shown in dendrogram graphics using the Ward Linkage method (Figure 5).

## Discussion

We performed reference charts for the fetal biometric parameters of the population in Karaman, a city in the Central Anatolia region, Turkiye. Some studies revealed that fetal biometric parameters can be changed with ethnic origin. Fetal biometry studies of different ethnic groups, including data from Belgium [8], Morocco [8], the United Kingdom [9], India [10], different ethnic groups in the USA [11], Brazil [12], Burkina Faso [13], China [14], France [15], Sudan [16], Nigeria [17, 18], Poland [19], and Italy [20] were performed in the past. There are several studies on fetal biometry for people living in Turkiye [21-28]. However, there is no standard chart of each fetal biometric parameter for the Turkish population, especially for the Central Anatolian population.

Ziylan et al. published a study regarding different femoral growth parameters in Central Anatolia, including femur length, on 30 fetal cadavers in 2003 [28]. They concluded that the measurement of fetal femur length can be considered one of the most reliable methods for assessing gestational age.

Malas et al. [25] studied 235 fetal cadavers who were aborted or died between six and 40 weeks of gestation to determine the fetal age according to different parameters. They determined that there were differences between the gestational ages of each fetal parameter. The correlation coefficients of gestational ages according to fetal parameters were different between trimester groups.

Varol et al. collected ultrasonographic data, including BPD, AC, and FL, from 1411 fetuses in 2001 (Trakya, Turkiye) [27]. The correlation between gestational age and these parameters was shown with graphics in their study. They gave the results of their data as mean dimensions by percentiles without gestational week (between 13 and 40 weeks). Therefore, these results cannot be practically applicable to routine measurements, and we could not match our results with them.

Demircan et al. [23] planned a longitudinal study and measured the ultrasonographic BPD, HC, AC, FL, and humerus length of 30 fetuses during gestation (between 14 and 40 weeks with two-week intervals). They represented their results for each parameter biweekly and compared them with other studies. They concluded that their nomograms correlate well with the work of Hadlock et al.

Basbug et al. measured the ultrasonographic abdominal circumference of 1038 fetuses between 12 and 41 gestational weeks [21]. They represented their results as percentile tables for each gestational week and compared other studies. They found significant differences in the results of abdominal circumference dimensions between national [21, 26, 29] and other studies on different ethnic origins. So they concluded that national and even regional parameter charts are important for routine clinical use. The studies of Basbug et al. [21], Sener et al. [26], and Ozgunen et al. [29], which were conducted in locations of Central Anatolia (Kayseri, Eskisehir, and Cukurova, respectively), had substantially similar results to our study; as these studies took place in locations close to our city, these similarities were in line with our expectations. Sener et al. [26] recorded BPD, AC, and FL measurements in percentiles between 20 and 41 weeks of gestation. However, Basbug et al. [21] only had AC measurements.

Ziylan et al. [28] and Malas et al. [25] performed their study on fetal cadavers. Varol et al. [27] gave the results of their data (BPD, AC, FL) as mean dimensions by percentiles without gestational week. So we could not make any comparison with these studies. Demircan et al. [23] performed a longitudinal study of only 30 fetuses. Basbug et al. [21] gave measurements only for abdominal circumference. Only Ozgunen et al. [29], and Sener et al. [26] had BPD, AC, and FL measurements by percentiles. These are a few studies published regarding BPD, HC, and FL measurements for the Turkish population. We compared our results with their results.

Louis et al. designed a cohort study for fetal growth standards in US ethnic groups, including non-Hispanic whites, non-Hispanic blacks, Hispanics, and Asians. Humerus, FL (10 weeks), AC (16 weeks), HC (21 weeks), and BPD (27 weeks) measurements were found to be significantly different among these four groups. EFW differed significantly by ethnicity after 20 weeks of gestation [6].

Tamura et al. [30] had fetal AC measurements of 197 fetuses. However, they stated that the group consists of heterogenous ethnic origin pregnant women. So these data are not suitable for ethnicity assessment.

Salomon et al. advised that the relevance of fetal measurements with expected values calculated upon reference equations used in each institution should be controlled. They suggested that the application of Z-scores allows for more accurate use of reference charts [2].

Peixoto et al. from Brazil made reference charts including 5th, 50th, and 95th percentiles and also made percentile graphics [12]. Leung et al., from Hong Kong, China, analyzed fetal biometric measurements from 709 singleton pregnancies. They suggest a new equation for fetal biometry and present their fetal percentile graphics [14].

Maternal factors that are effective in fetal biometry were investigated in some studies [10, 11]. Tarca et al. suggest that maternal height had a significant effect on all centiles of EFW, yet the effect was higher for the most extreme centiles. The effect of maternal weight on all centiles of EFW at 40 weeks was up to a 1.4% increase for each additional 10 kg in maternal weight. They also determined that the fetuses of parous women had a higher EFW than those of nulliparous women, although the magnitude of such an effect varied among centiles and changed with gestational age [11]. They found that the EFW of male fetuses was about 2% higher than that of female fetuses, independent of all other factors. Bad habits like smoking also distinctly affect babies' development, especially their birth weight [10, 11].

We compared our study results with other ethnic populations (non-Hispanic White, Asian, Brazilian, Polish, Nigerian, Italian, American) results [6, 12, 17, 19, 20, 30]. Our studies' BPD, HC, AC, and FL values have statistically similar means in all studies according to Kruskal-Wallis test results. However, there were statistically significant results with hierarchical cluster analysis. Analysis of our study's results has found similar results to Italian populations' results for BPD, HC, AC, and FL percentile curves. Our all percentile curves results were far apart from Asia populations up to the last trimester of gestation.

As per the content of the title and content of this paper, we conducted this study on a Central Anatolian Turkish population. This study does not evaluate the whole Turkish population; our comparisons were made between a Central Anatolia Turkish population and other ethnic populations. Similarly, the study from Brazil [12] studied a population from the "metropolitan region of Uberaba, Minas Gerais state, Southeast of Brazil", not the whole of Brazil. The study from Italy [20] studied a population in a single center; i.e. the Artemisia Fetal Maternal Medical Centre. Comparisons within this study are just between study groups.

We chose the single-center structure for our study to maintain constant population sensitivity. However, multicenter studies may have more participation. Also, similar studies can be conducted across different regions of Turkiye, allowing for countrywide comparisons.

## Conclusions

The assessment of fetal growth rate and the specialization of fetal biometric charts for a particular population can improve the possibility of detecting high-risk fetuses. Fetal biometric percentile tables and graphics of a Central Anatolia Turkish population were represented in this study. Our study results have been found closer to Italian population results for BPD, HC, AC, and FL percentile curves, probably resulting from geographical promixity. However, Asian and Brazilian populations had very different results. Our study's data will provide a valuable contribution to obstetrical practice.

## References

[1] Abuhamad A (ed) (2014). Ultrasound in Obstetrics and Gynecology: A practical approach. Ultrasound in Obstetrics and Gynecology: A practical approach.

[2] Salomon LJ, Alfirevic Z, Da Silva Costa F (2019). ISUOG Practice Guidelines: ultrasound assessment of fetal biometry and growth. Ultrasound Obstet Gynecol.

[3] Hadlock FP, Deter RL, Harrist RB, Park SK (1984). Estimating fetal age: computer-assisted analysis of multiple fetal growth parameters. Radiology.

[4] Hadlock FP, Harrist RB, Sharman RS, Deter RL, Park SK (1985). Estimation of fetal weight with the use of head, body, and femur measurements--a prospective study. Am J Obstet Gynecol.

[5] March MI, Warsof SL, Chauhan SP (2012). Fetal biometry: relevance in obstetrical practice. Clin Obstet Gynecol.

[6] Buck Louis GM, Grewal J, Albert PS (2015). Racial/ethnic standards for fetal growth: the NICHD Fetal Growth Studies. Am J Obstet Gynecol.

[7] Salomon LJ, Alfirevic Z, Berghella V (2011). Practice guidelines for performance of the routine mid-trimester fetal ultrasound scan. Ultrasound Obstet Gynecol.

[8] Jacquemyn Y, Sys SU, Verdonk P (2000). Fetal biometry in different ethnic groups. Early Hum Dev.

[9] Altman DG, Chitty LS (1997). New charts for ultrasound dating of pregnancy. Ultrasound Obstet Gynecol.

[10] Aggarwal N, Sharma GL (2020). Fetal ultrasound parameters: Reference values for a local perspective. Indian J Radiol Imaging.

[11] Tarca AL, Romero R, Gudicha DW (2018). A new customized fetal growth standard for African American women: the PRB/NICHD Detroit study. Am J Obstet Gynecol.

[12] Peixoto AB, da Cunha Caldas TM, Dulgheroff FF, Martins WP, Araujo Júnior E (2017). Fetal biometric parameters: Reference charts for a non-selected risk population from Uberaba, Brazil. J Ultrason.

[13] Bihoun B, Zango SH, Traoré-Coulibaly M (2020). Fetal biometry assessment with Intergrowth 21st's and Salomon's equations in rural Burkina Faso. BMC Pregnancy Childbirth.

[14] Leung TN, Pang MW, Daljit SS, Leung TY, Poon CF, Wong SM, Lau TK (2008). Fetal biometry in ethnic Chinese: biparietal diameter, head circumference, abdominal circumference and femur length. Ultrasound Obstet Gynecol.

[15] Salomon LJ, Duyme M, Crequat J, Brodaty G, Talmant C, Fries N, Althuser M (2006). French fetal biometry: reference equations and comparison with other charts. Ultrasound Obstet Gynecol.

[16] Ayad CE, Ibrahim AA, GarElnabi ME, Ahmed BH, Abdalla EA, Saleem MA (2016). New Sudanese reference chart of fetal biometry and weight using ultrasonography. Open J Radiol.

[17] Adiri CO, Anyanwu GE, Agwuna KK (2015). Use of fetal biometry in the assessment of gestational age in South East Nigeria: Femur length and biparietal diameter. Niger J Clin Pract.

[18] Mador ES, Pam IC, Ekedigwe JE (2012). Ultrasound biometry of Nigerian fetuses: 2. Femur length. Asian J Med Sci.

[19] Dubiel M, Krajewski M, Pietryga M, Tretyn A, Breborowicz G, Lindquist P, Gudmundsson S (2008). Fetal biometry between 20-42 weeks of gestation for Polish population. Ginekol Pol.

[20] Giorlandino M, Padula F, Cignini P, Mastrandrea M, Vigna R, Buscicchio G, Giorlandino C (2009). Reference interval for fetal biometry in Italian population. J Prenat Med.

[21] Basbug M, Aygen E, Serin S, Tayyar M (2000). Fetal biometry: Abdominal circumference percentiles [article in Turkish]. J Clin Pract Res.

[22] Bese T, Yalcinkaya T, Demir F (1995). Ultrasonogram ile tepe-makat uzunlugu, biparietal cap, fronto-oksipital cap, kafa cevresi, abdominal cevre ve femur uzunluğu olcumlerine ait nomogramlar [article in Turkish]. Perinatoloji Dergisi.

[23] Demircan A, Berkol Y (1988). Growth curves derived from ultrasonographic fetal parameters in a Turkish population. Marmara Med J.

[24] Kabaalioglu A (2017). İkinci trimester ultrasonografi incelemesi [article in Turkish]. Trd Sem.

[25] Malas MA, Desdicioglu K, Cankara N, Evcil E, Özgüner G (2007). Fetal dönemde fetal yaşın belirlenmesi [article in Turkish]. Med J SDU.

[26] Sener T, Hassa H, Tekin B, Ramazan Ramazan, Bayırlı R, Bal CZ (1996). Are fetal growth nomograms different at Osmangazi University obstetrics population? [article in Turkish]. T Klin Jinekol Obst.

[27] Varol F, Saltik A, Kaplan PB, Kiliç T, Yardim T (2001). Evaluation of gestational age based on ultrasound fetal growth measurements. Yonsei Med J.

[28] Ziylan T, Murshed KA (2003). An assessment of femur growth parameters in human fetuses and their relationship to gestational age. Turk J Med Sci.

[29] Ozgunen T, Evruke İC, Atay Y (1996). Cukurova yoresinde normal gebe populasyonunda ultrasonografik fetal biometri [article in Turkish]. Medical Network Klinik Bilimler Kadın Doğum Dergisi.

[30] Tamura RK, Sabbagha RE, Pan WH, Vaisrub N (1986). Ultrasonic fetal abdominal circumference: comparison of direct versus calculated measurement. Obstet Gynecol.

